# Occupational Airborne Allergic Contact Dermatitis From UV‐Curing Printing Varnish

**DOI:** 10.1111/cod.70115

**Published:** 2026-03-02

**Authors:** Magalie Coco‐Viloin, Caroline Gernigon, Fabrice Herin

**Affiliations:** ^1^ Department of Occupational and Environmental Disease Purpan Hospital Toulouse France

**Keywords:** acrylates, airborne allergic contact dermatitis, case report, occupational, patch test, UV‐curable ink

Acrylates have repeatedly been reported as sensitizers in ultraviolet‐curable ink. We report a case of severe occupational airborne allergic contact dermatitis caused by acrylate‐containing varnish.

## 1 Case Report

**FIGURE 1:**
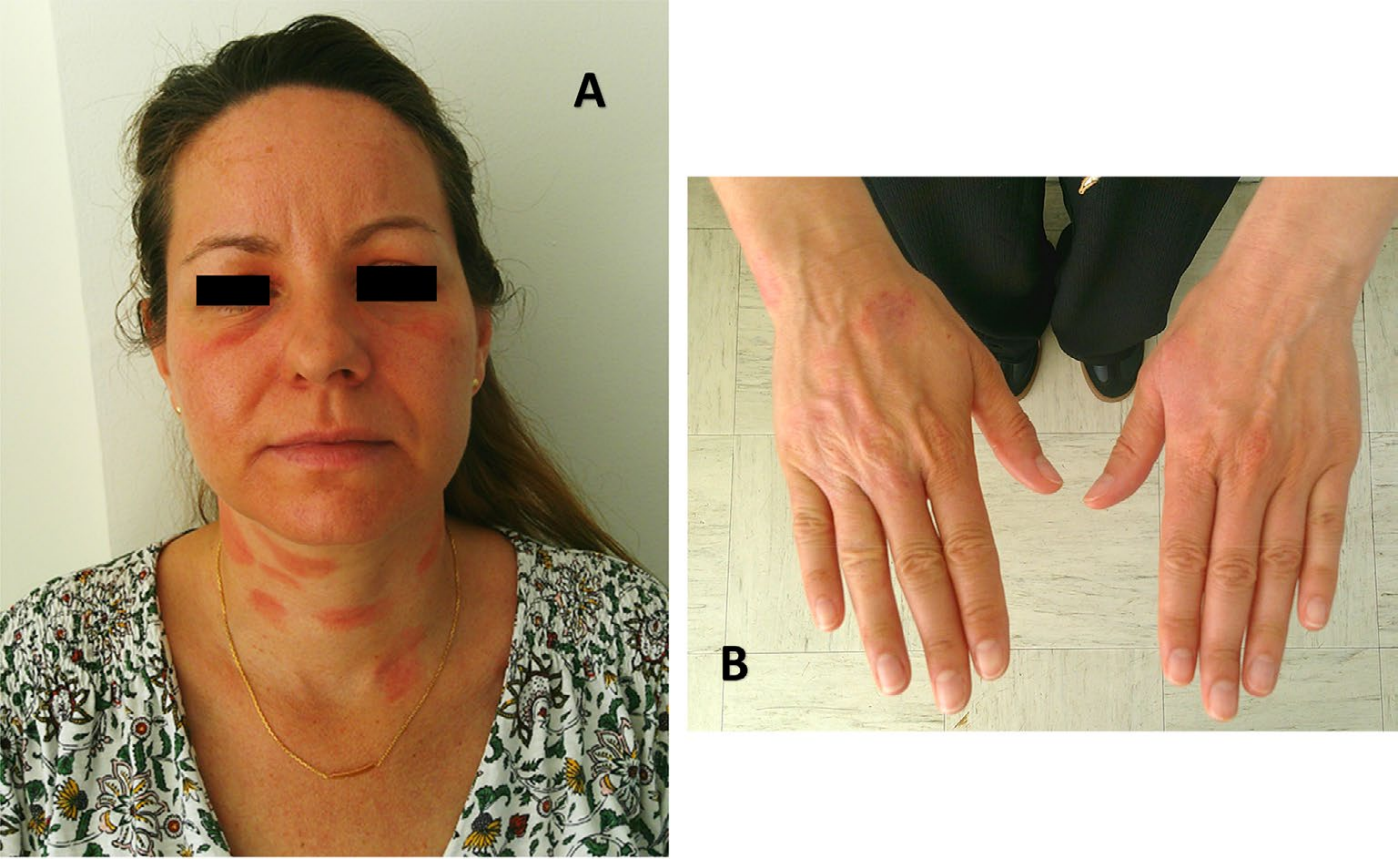
(A) Severe eczema on the face and neck. (B) Eczema on the dorsal surfaces of the hands.

A 44‐year‐old woman with a history of atopy, including atopic dermatitis and respiratory allergy to pollen was referred to the dermatology and allergology department for a severe skin eruption. In December 2024, she developed a lesion on her upper eyelids, which subsequently became bilateral, affecting both upper and lower eyelids. In the following months, erythematous eruption appeared progressively on the dorsal surfaces of the hands, anterior wrists and anterior cervical region. Treatment with desonide 0.05% was effective on the face; however, the lesions relapsed shortly after discontinuation. By May 2025, the clinical presentation was suggestive of airborne allergic contact dermatitis (Figure 1).

During the clinical interview, several potential contact allergens were identified, appearing after a relocation in November 2024 involving construction work and the use of paints. The patient also reported exposure to household detergents, essential oil‐based products, cosmetics, and makeup. Additionally, she had recently switched to photopolymerizable ink and varnish at her workplace, where she had been working as a graphic designer for 3 years, creating signs, flyers and posters. However, the workplace exemption test was negative, likely because she had only been off work for 1 week.

The exact composition of the ink was initially unknown, but acrylates were suspected due to the light‐curing properties reported by the patient. Given the high prevalence of allergic contact dermatitis in the printing industry, an occupational origin was strongly suspected—especially considering the airborne distribution of the rash and the well‐known potential for acrylates to cause allergic contact dermatitis [1].

**FIGURE 2:**
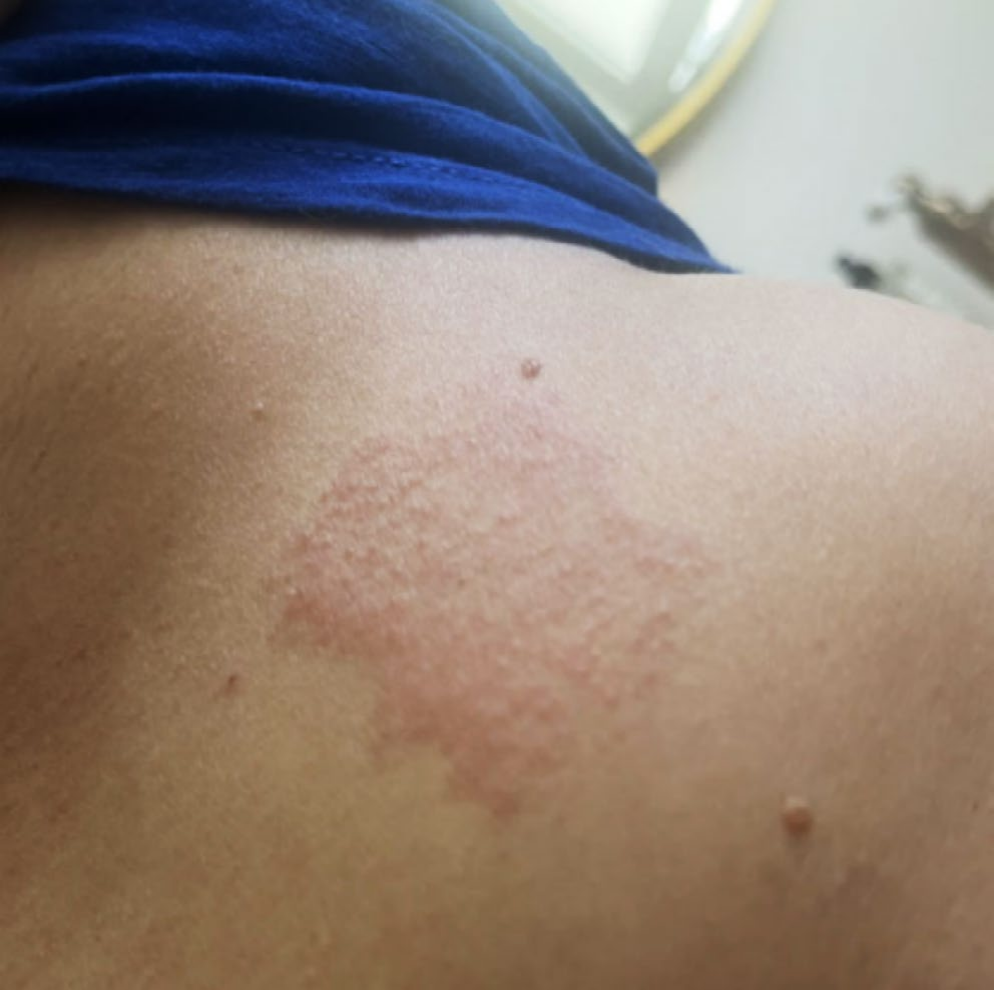
Positive semi‐open patch test with UV‐curable ink as is at Day 2, self‐applied by the patient.

Following the initial consultation, the patient independently performed a semi‐open test with UV ink as is applied directly on her belly for 2 h. On Day (D) 2, a non‐irritant positive reaction was observed (Figure 2). Patch tests were performed with the European baseline series, cosmetic series, acrylate series and epoxy series (Chemotechnique Diagnostics, Vellinge, Sweden), varnish 1% petrolatum (pet.) and ink 1% pet according to published recommendations [2]. Patch test chambers used were IQ ultra (Chemotechnique Diagnostics, Vellinge, Sweden) for 2 days. Readings performed on D2 and D3 (Table 1).

**TABLE 1:** Patch test results on Day 2 (D2) and Day 3 (D3).

	D2	D3
European baseline series	Nickel sulfate 5% pet (++) Myroxylon *pereirae* 25% pet (+)	Nickel sulfate 5% pet (++) Myroxylon *pereirae* 25% pet (++)
Cosmetic series	Negative	Negative
Acrylate series	1,6‐Hexanediol diacrylate 0.1% pet (+)	1,6‐Hexanediol diacrylate 0.1% pet (+)
Epoxy series	Negative	Negative
*Varnish 1% petrolatum (pet.)*	(?+)	(+)
*Ink 1% pet*	(?+)	(+)

*Note*: Products shown in italics were supplied by the patient.

**FIGURE 3:**
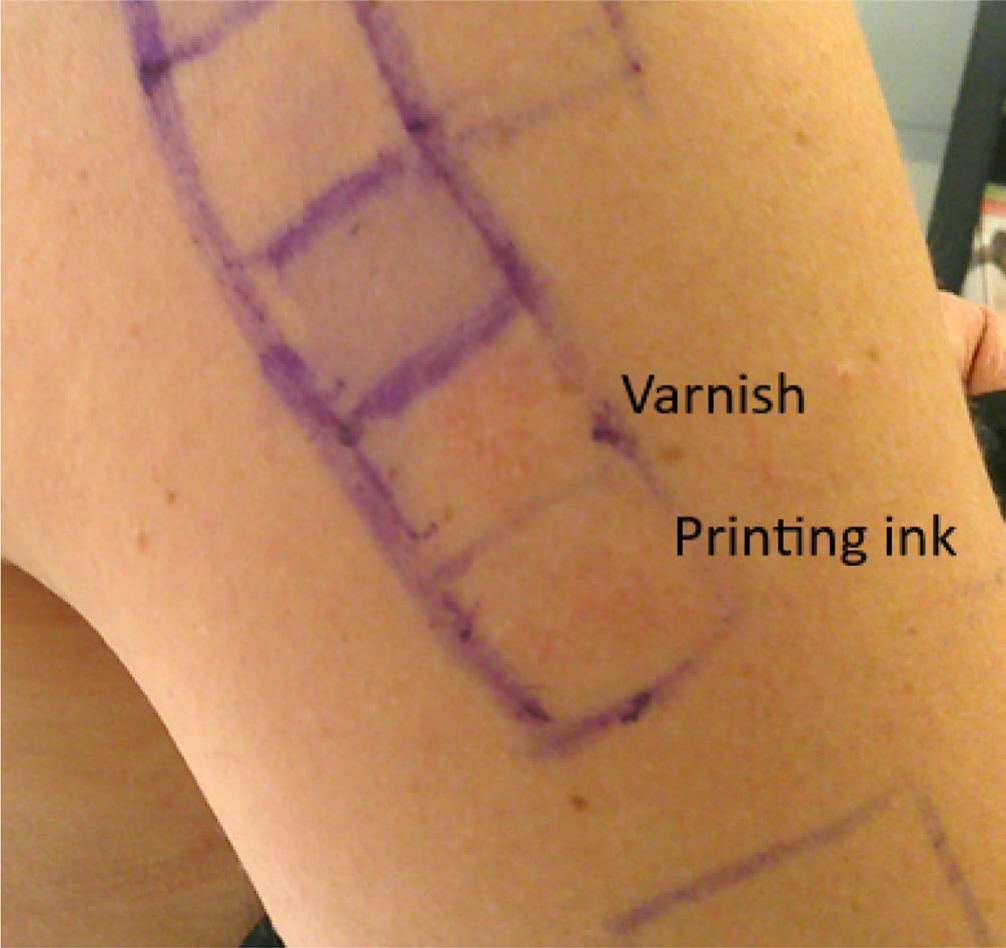
Positive (+) patch test on Day 3 for varnish (1% in petrolatum) and printing ink (1% in petrolatum).

On D3, patch tests were positive (+) for varnish, ink (Figure 3) and 1,6‐Hexanediol diacrylate 0.1% pet. Patch test with nickel sulphate was also positive (++), with former relevance due to prior reactions to costume jewellery. Patch test with *Myroxylon pereirae* was positive (++) but its relevance was unknown. The material safety data sheet collected afterwards confirmed the presence of 1,6‐Hexanediol diacrylate in the varnish (Additional File: produit‐uv‐ink and produit‐varnish), confirming the diagnosis of occupational airborne allergic contact dermatitis.

Following the tests, the patient underwent a work leave, resulting in complete healing of the lesions after 3 weeks. A certificate of occupational disease was issued according to Table no. 65 of the French social security system.

## 2 Discussion

Acrylates are widely used in the printing industry as monomers and prepolymers in UV‐curable inks and overprint varnishes. Their rapid polymerisation under UV light makes them highly effective for fast‐drying applications. Several cases of contact allergy to acrylates in this industry have been reported [3, 4, 5, 6, 7].

In our case, 1,6‐Hexanediol diacrylate (1,6‐HDDA), a highly sensitising diacrylate monomer, was identified as the culprit allergen in the varnish. This finding aligns with prior reports highlighting HDDA as a frequent allergen in UV‐curable products [7]. While the specific allergen responsible in the ink was not definitively identified, suspicion fell on 1,6 HDDA due to the presence of acrylic ester listed in the material safety data sheet. This underscores the challenge of identifying causative agents in complex industrial mixtures, where patch test with relevant workplace materials is critical. Interestingly, no cross‐reactivity was observed among the various acrylates tested in our patient.

It is also important to note that 1,6‐HDDA is found in coatings, adhesives, health products, and clothing materials, which broadens the range of potential sensitization sources [8, 9, 10]. In our patient's case, sensitization was occupational, with no other sources of exposure identified.

A limitation of our case report is that, although a 1% dilution of the patient's own product was used, no dilution series or control group testing was performed. Additionally, the absence of a Day 7 reading should be noted, as such measures are important to exclude irritant reactions and detect delayed onset reactions.

Finally, the short duration of work absence was insufficient for complete healing of the lesions. This case illustrates the importance of testing with the workplace allergens to guide appropriate management.

## Author Contributions

**Magalie Coco‐Viloin:** conceptualization, investigation, writing – original draft, writing – review and editing. **Caroline Gernigon:** review. **Fabrice Herin:** review and validation.

## Conflicts of Interest

The authors declare no conflicts of interest.

## References

[1] S. Huygens and A. Goossens , “An Update on Airborne Contact Dermatitis,” Contact Dermatitis 44, no. 1 (2001): 1–6. 11156004 10.1034/j.1600-0536.2001.440101.x

[2] A. C. De Groot , Patch Testing Four Edition (Acdegroot Publishing, 2018).

[3] K. Aalto‐Korte and K. Suuronen , “Ten Years of Contact Allergy From Acrylic Compounds in an Occupationaldermatology Clinic,” Contact Dermatitis 84 (2021): 240–246. 33184864 10.1111/cod.13739

[4] H. Wahlkvist and A.‐C. Kaaman , “Occupational Contact Allergy to 2‐Butylaminocarbonyloxyethylacrylate in UV‐Curing Printing Inks,” Contact Dermatitis 82 (2020): 325–326. 31951281 10.1111/cod.13471

[5] E. Higgins and P. Collins , “Urticarial Allergic Contact Dermatitis Caused by UV‐Cured Printing Ink,” Contact Dermatitis 66, no. 6 (2012): 340–341. 22568841 10.1111/j.1600-0536.2012.02010.x

[6] R. J. Rycroft , “Occupational Contact Dermatitis,” in Textbook of Contact Dermatitis, 2nd ed., ed. R. J. Rycroft , T. Menné , and P. Frosch (Springer‐Verlag, 1999), 343–400.

[7] V. A. Morgan and J. M. Fewings , “1,6‐Hexanediol Diacrylate: A Rapid and Potent Sensitizer in the Printing Industry,” Australasian Journal of Dermatology 41, no. 3 (2000): 190–192. 10954995 10.1046/j.1440-0960.2000.00429.x

[8] N. Raison‐Peyron , A. Poncy , J. Dahlin , and C. Svedman , “Allergic Contact Dermatitis to 1.6‐Hexanediol Diacrylate in Ski Boots,” Contact Dermatitis 93 (July 2025): 527–529. 40730388 10.1111/cod.70005 PMC12586287

[9] A. Badaoui , “Allergic Contact Dermatitis to 1,6 Hexanediol Diacrylate: A New Allergen in Diabetes Devices?,” Contact Dermatitis 93, no. 1 (July 2025): 66–67. 40098457 10.1111/cod.14778

[10] I. Siemund , J. Dahlin , M. Mowitz , N. Hamnerius , and C. Svedman , “Allergic Contact Dermatitis due to 1,6‐Hexanediol Diacrylate in Ostomy Patients,” Contact Dermatitis 90, no. 5 (May 2024): 501–506. 38332444 10.1111/cod.14516

